# WiggleTools: parallel processing of large collections of genome-wide datasets for visualization and statistical analysis

**DOI:** 10.1093/bioinformatics/btt737

**Published:** 2013-12-19

**Authors:** Daniel R. Zerbino, Nathan Johnson, Thomas Juettemann, Steven P. Wilder, Paul Flicek

**Affiliations:** European Molecular Biology Laboratory, European Bioinformatics Institute (EMBL-EBI), Wellcome Trust Genome Campus, Hinxton, Cambridge CB10 1SD, UK

## Abstract

**Motivation:** Using high-throughput sequencing, researchers are now generating hundreds of whole-genome assays to measure various features such as transcription factor binding, histone marks, DNA methylation or RNA transcription. Displaying so much data generally leads to a confusing accumulation of plots. We describe here a multithreaded library that computes statistics on large numbers of datasets (Wiggle, BigWig, Bed, BigBed and BAM), generating statistical summaries within minutes with limited memory requirements, whether on the whole genome or on selected regions.

**Availability and Implementation:** The code is freely available under Apache 2.0 license at www.github.com/Ensembl/Wiggletools

**Contact:** zerbino@ebi.ac.uk or flicek@ebi.ac.uk

## 1 INTRODUCTION

With the advent of high-throughput sequencing, research teams and consortia are generating large numbers of datasets that are projected onto the same reference genome (Adams *et al.*, 2012; Bernstein *et al.,* 2010; The ENCODE Project Consortium, 2012). In particular, epigenomic assays quantify many continuous variables across the genome, e.g. transcription factor binding, histone marks, DNA methylation, chromatin structure or RNA transcription.

Although they differ in their protocols, all the above assays include a sequencing step that generates a huge number of sequencing reads. These reads, or tags, are then aligned against the human genome. This placement information is normally stored in the BAM file format (Li *et al.*, 2009). Because the BAM files are generally large and information rich, they are often summarized into BigWig files that describe a numerical variable such as read depth across the genome (Kent *et al.*, 2010). These BAM and BigWig files can then readily be displayed on most genome browsers (Flicek *et al.*, 2013; Meyer *et al.*, 2013).

In the current context, where researchers are testing many measurements across many samples, displaying all these data creates confusing graphics: either the plots are placed side-by-side and an observer is forced to continually shift their attention from one plot to another, or the plots are superimposed, blurring the information content.

Instead, one could summarize all these datasets for each position in the genome. Similarly, one could display the difference between case and control datasets. Fundamentally, all of these datasets are simply vectors of numbers, and statistics, such as mean, variance, median, etc., can be generated from any such collection, producing a meaningful summary of the data.

Common statistical tools such as *R* (*R* Core Team, 2013) do not scale well to such large datasets, especially with respect to memory requirements. Therefore, we developed a tool that can perform rigorous statistical tests across the whole genome and detect regions of interest without practical memory constraints.

We drew inspiration from the popular BEDTools package (Quinlan and Hall, 2010), which computes overlaps and derived statistics between sets of regions. Converting numerical measurements into genomic regions (generally referred to as peak calling or segmentation, depending on the context) is a convenient and common approach to handling genome-wide data. However, it does imply an inevitable loss of information, as continuous variables are discretized and often binarized. Therefore, we wanted a tool that natively reads the numerical data contained in genomic files and computes statistics on it.

## 2 FEATURES AND METHODS

### 2.1 Composable iterators

WiggleTools is centered on the use of iterators. This approach ensures scalability and reduces memory requirements: instead of loading entire files in memory, an iterator simply stores local information, allowing a program to simultaneously process dozens, even hundreds of files. This simultaneous handling of multiple files is particularly useful to compute statistics such as medians, which require storing all possible values before evaluation. The only exceptions to the use of iterators are the input/output operations, which are run on separate threads that read/write, compress/decompress and parse/print data files independently.

The basic iterators simply read the data from files, whether BAM, Wiggle, BigWig, Bed, BigBed or BedGraph. A range of iterators can be built on top of those. There are basic unary operators (multiplication by a constant scalar, absolute value, logarithm, exponential, exponentiation and filter), binary operators (sum, product, ratio and difference), statistics on sets (mean, median, standard deviation, variance, minimum, maximum) and statistics on pairs of sets (Welch’s *t* test, Mann–Whitney U). In turn, all these iterators can be combined or composed to create more complex operators. Iterators can either traverse the entire genome or a slice of the genome.

### 2.2 Functionalities

The primary intent of the library is to compute statistics across a large number of datasets, so that the users need only display one curve on their genome browser instead of a multitude. For example, they can compress a collection of datasets into a median, as well as compare datasets (e.g. cases versus controls) and generate a track that denotes the differences between the two sets.

In addition, the WiggleTools library can compute statistics across genomic positions for a single iterator (area under the curve, variance) or a pair of iterators (Pearson correlation). These statistics can be computed across the entire genome or on regions of interest. For example, it can compute the read coverage at known promoter regions. Similarly, WiggleTools can be used to compute a scaled summary profile of the data on a set of regions.

The WiggleTools library can be used as a C library but also as a standalone command-line tool. The user has complete access to the richness of the framework using a simple Polish Notation language. For example, to generate the sum of a collection of BigWig files and write the result into a new Wiggle file, the command would look like:

wiggletools write sum.wig sum data/*.bw

### 2.3 Performance

The WiggleTools library has been specifically designed to handle many files simultaneously, allowing complex statistics to be computed as directly as possible, with low memory requirements. The limiting factor of this approach is the I/O access to the files, meaning that it requires the input files to be in the local network of the computation CPUs. However, because of the efficient indexing of BigWig files, the output can be directly displayed on a remote server, such as a genome browser.

It is trivial to accelerate computations by slicing the genome into regions and assigning each region to a different CPU. A wrapper script is available to do this automatically. However, one obstacle to this approach is merging the final files, as the tools provided in the original Kent library quickly become a performance bottleneck. Therefore, we developed modified functions that parallelize the computation of summary tables (which are crucial to accelerate display at large scales), which we contributed to the Kent library.

To evaluate the performance of our tool, we downloaded all the DNAseI hypersensitivity wiggle tracks contained on the ENCODE January 2011 data freeze (The ENCODE Project Consortium, 2012) and computed the sum of all these signals through three pipelines. We first ran the WiggleTools library in parallel on 116 sections of the genome (up to 30-Mbp long), producing as many output BigWig files that were merged with our new bigWigCat utility. Second, we ran WiggleTools but merged the output files with the default bigWigMerge utility (Kent *et al.*, 2010). Finally, we used bigWigMerge to directly sum the 126 BigWig files. The bigWigMerge tool only creates flat files; therefore, a compression and indexing stage, performed by the wigToBigWig tool, must also be done. The results in Table 1 clearly show that the first pipeline, which took 1090s to run, is ∼12 and 19 times faster than the other approaches, while requiring a fraction of the memory.

**Table 1.** Benchmarking CPU and memory requirements to compute the sum of 126 BigWig files (121 GB of data in total)

Pipeline	Stage	CPUs	Time/CPU (s)	RAM/CPU (GB)
1	wiggletools	116	351 mean	0.22 mean
			739 maximum	0.32 maximum
	bigWigCat	1	378	5.23
	Overall	116	1090	5.23
2	wiggletools	116	351 mean	0.22 mean
			739 maximum	0.32 maximum
	bigWigMerge	1	3441	6.93
	wigToBigWig	1	8887	68.85
	Overall	116	13 067	68.85
3	bigWigMerge	1	11 036	43.73
	wigToBigWig	1	9423	75.12
	Overall	1	20 459	75.12

*Note*: Several pipelines are compared; hence some components appear multiple times.
